# Topological and Reconfigurable Terahertz Metadevices

**DOI:** 10.34133/research.0882

**Published:** 2025-08-27

**Authors:** Zihan Zhao, Hongwei Wang, Guangwei Hu, Andrea Alù

**Affiliations:** ^1^ School of Electrical and Electronic Engineering, 50 Nanyang Avenue, Nanyang Technological University, Singapore 639798, Singapore.; ^2^ Photonics Initiative, Advanced Science Research Center, City University of New York, New York, NY 10031, USA.; ^3^ Physics Program, Graduate Center of the City University of New York, New York, NY 10016, USA.

## Abstract

The terahertz (THz) frequency range, situated between microwave and infrared radiation, has emerged as a pivotal domain with broad applications in high-speed communication, imaging, sensing, and biosensing. The development of topological THz metadevices represents a notable advancement for photonic technologies, leveraging the distinctive electronic properties and quantum-inspired phenomena inherent to topological materials. These devices enable robust waveguiding capabilities, positioning them as critical components for on-chip data transfer and photonic integrated circuits, particularly within emerging 6G communication frameworks. A principal advantage resides in the capacity to maintain low-loss wave propagation while effectively suppressing backscattering phenomena, a critical requirement for functional components operating at higher frequencies. In parallel, by leveraging advanced materials such as liquid crystals, plasma, and phase-change materials, these devices facilitate real-time control over essential wave parameters, including amplitude, frequency, and phase, which augments the functionality of both communication and sensing systems, opening new avenues for THz-based technologies. This review outlines fundamental principles of topological components and reconfigurable metadevices operating at THz frequencies. We further explore emerging strategies that integrate topological properties and reconfigurability, with a specific focus on their implementation in chip-scale photonic circuits and free-space wavefront control.

## Introduction

Terahertz (THz) radiation uniquely bridges the gap between microwave and infrared regimes, offering non-ionizing, broadband capabilities ideal for next-generation sensing, imaging, and communication technologies [1–3]. Additionally, the strong interaction between THz waves and polar molecular species, such as aqueous solutions, protein structures, and explosive compounds, has established THz spectroscopy as a powerful tool for pharmaceutical quality control and biomedical diagnostics [4,5]. In wireless communications, the THz spectrum offers substantially broader bandwidth compared to conventional microwave frequencies, which is critical for advancing ultra-high-speed data transmission technologies in next-generation networks [6]. Moreover, THz-based remote sensing systems have been utilized for atmospheric monitoring and environmental analysis, providing high-resolution spectral data for molecular fingerprinting [7–9]. Despite these promising attributes, the inherently weak light–matter interaction in natural materials at THz frequencies presents a technological barrier.

Metadevices have emerged as an effective strategy to overcome intrinsic limitations of natural materials in the THz frequency range [10,11]. By tailoring the geometry and arrangement of meta-atoms, these engineered structures enable precise control over the amplitude, phase, polarization, and frequency of THz waves. Building on this capability, THz metadevices have been developed for a wide range of applications, including imaging [12], communication [13], and biosensing [14,15]. Continued advances in nanofabrication techniques and a deeper understanding of light–matter interactions at the nanoscale have further accelerated the development of THz metadevices.

The integration of topological physics with THz metadevices has opened new avenues for both on-chip and free-space applications. Topological insulators and 2-dimensional topological semimetals, characterized by their unique electronic band structures and protected surface states, are well suited for THz wave detection and control [16]. In on-chip scenarios, topological metadevices enable robust, low-loss THz wave propagation with suppressed backscattering [17], which is essential for THz photonic integrated circuits and future wireless communication systems [18]. For free-space applications, topological metadevices offer functionalities such as modulation [19], thereby expanding the capabilities of THz technologies across diverse domains. A breakthrough on the calibration-free THz diagnostic method was originated by dividing 2 transient-generated spectral lines with different resonance frequencies [20]. Pioneering studies on qualitative fingerprint recognition and quantitative sensing were created by metasurface-excited surface waves propagating over a long range [21].

The growing demands of modern communication and sensing systems have heightened the need for reconfigurable THz metadevices. To overcome the limited tunability of natural materials and enable real-time adaptability, various approaches have been explored, including liquid crystal (LC)-based tunable THz metadevices [22], phase-change materials (PCMs) [23], and reconfigurable platforms integrated with plasmonic structures [24]. These reconfigurable systems enable dynamic control of THz wave properties, offering enhanced versatility for optical device applications and improving the performance of THz communication and sensing technologies [25].

This review contains an overview of recent advances in THz metadevices. The first section examines distinct properties and application scenarios of topologically inspired devices, including on-chip integration platforms and free-space implementations. The second section focuses on reconfigurable THz metadevices based on a range of functional materials, detailing their modulation mechanisms and representative applications. The third section explores the development of reconfigurable topological THz platforms and highlights their promising application potential. Finally, current challenges and future research directions for reconfigurable topological THz metadevices are discussed.

## Topological THz Metadevices

THz topological metadevices are subwavelength 2-dimensional artificial electromagnetic materials engineered with topological physics to enable robust wavefront control and lossless edge-state propagation of THz waves. Their unique structures allow for the manipulation of THz waves to enhance the absorption and conversion efficiency of THz radiation into electrical signals. In particular, this device can be incorporated into integrated circuits to manipulate and detect THz waves on a chip scale, facilitating the development of compact and high-performance THz communication and imaging systems. The meta-atoms in the metadevices can be designed to resonate over a wide range of THz frequencies, enabling the detection of a broad spectrum of THz waves.

### On-chip platforms

Recent advancements in on-chip topological THz metadevices are advancing THz system capabilities through robust wave manipulation enabled by topological photonics. Emerging strategies address critical challenges in THz technology: photonic supercoupling enables multi-wavelength waveguide-cavity energy transfer across extended distances [26], while topologically protected waveguide tapers demonstrate resilience to structural imperfections, establishing a robust platform for compact THz integrated circuits [27]. The integration of topological photonics with THz technology has also driven critical advances in communication and sensing systems. Quantum transport experiments reveal preserved frequency entanglement in valley photonic crystal waveguides, maintaining quantum correlations through sharp bends via the quantum valley Hall effect [28]. A chip-scale topological diplexer further demonstrates 150 Gbit/s operation through Klein tunneling-mediated THz band filtering [29]. In addition, robust topological valley transport in THz regimes has been achieved on an all-silicon platform, achieving error-free data transmission at 11 Gbit/s. This capability enables real-time uncompressed 4K video streaming and demonstrates the viability of topological approaches for high-speed THz communication systems [30]. For sensing applications, exploiting extended evanescent fields in topological cavity-waveguide hybrids to achieve precise detection of sub-wavelength frequency shifts for biomolecular analysis and plant hydration monitoring [31]. Groundbreaking methods on simultaneously recognizing diverse molecules were proposed [32] and demonstrated via landmark self-similar multi-resonant units with gradually changed resonant peaks to achieve independently regulated enhancement of multiple spectral frequencies [33]. An innovative ultra-sensitive THz metasensor was created by exploring quasi-bound states in the continuum combining gold nanoparticles conjugated with specific antibodies [34].

**Fig. 1.**
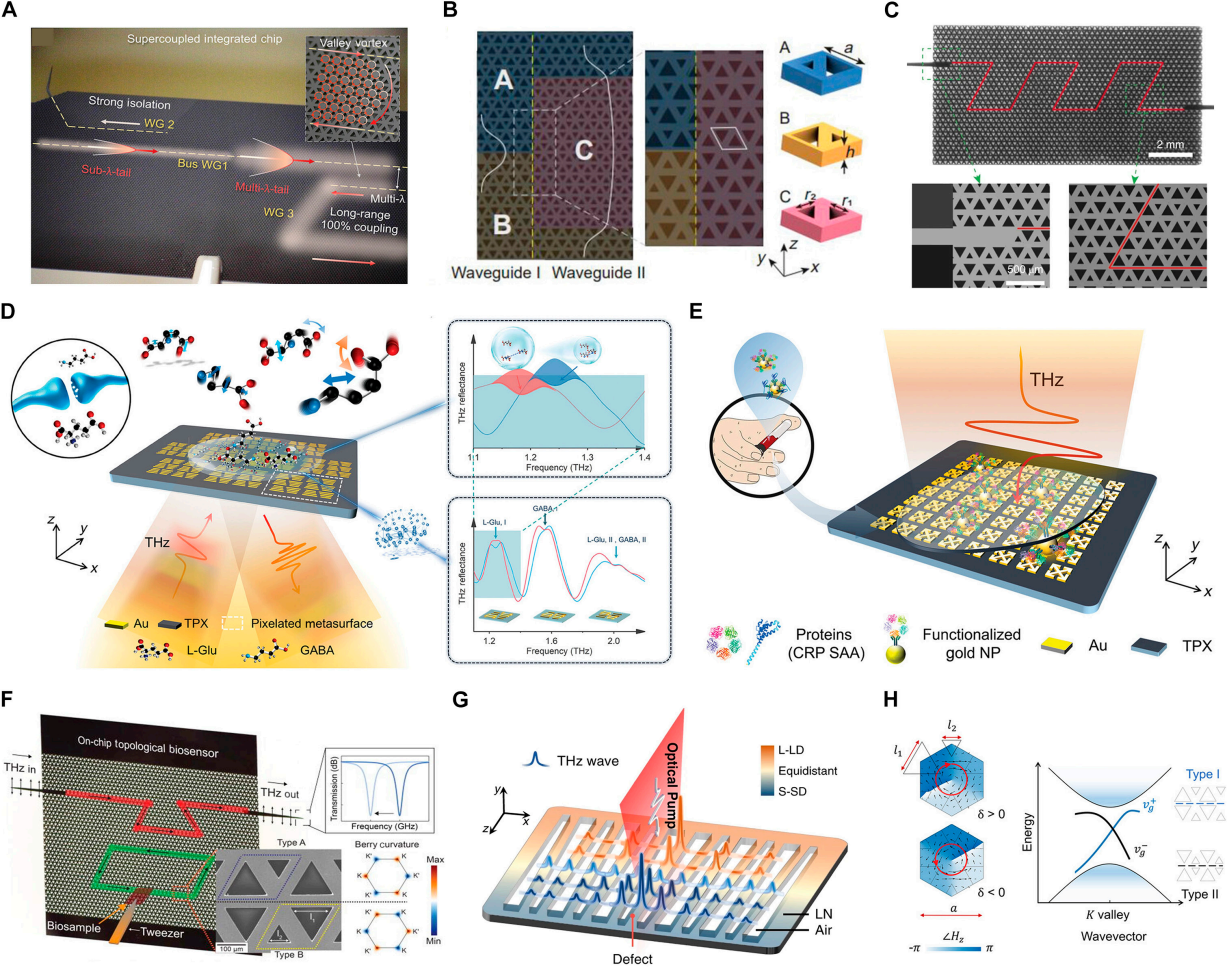
On-chip topological THz metadevices (A) Photonic supercoupling in silicon topological waveguides [26]. (B) On-chip THz taper-free waveguides [27]. (C) THz topological photonics for on-chip communication [30]. (D) Landmark self-similar multi-resonant units with gradually changed resonant peaks [33]. (E) Quasi-BIC-based metasensors [34]. (F) On-chip topological THz biosensors [31]. (G) Nonlinear generation and topologically tuned confinement of THz waves [36]. (H) On-chip active supercoupled topological cavity [37].

The on-chip topological THz metadevice further extends the functionality by integrating modulation and signal processing modules. For example, metallic quantum well-based topological modulators demonstrate enhanced bandwidth and modulation depth in compact configurations [35]. Engineered lithium niobate chips achieve precise THz wave confinement through topological tuning [36]. Moreover, photonic supercoupling achieves 91% efficiency across multi-wavelength separations while maintaining 30 dB isolation [37]. In summary, on-chip topological THz devices achieve multi-wavelength long-distance energy transmission and defect-resistant waveguide transmission through photonic supercoupling and topological protection mechanisms, greatly improving the stability and integration of the system. In particular, metadevices achieved using complementary metal oxide semiconductor (CMOS) technology can integrate transistors so that the transistors can be used to dynamically control the switching of the array of metadevices and to control the beamforming characteristics in real time. In the future, it will show application advantages in key applications such as communications, quantum frequency entanglement maintenance, and high-sensitivity detection of biomolecules.

### Free-space applications

THz topological metadevices also provide solutions to practical challenges in free-space systems. Research on topological photonic crystals has been revealing simultaneous support of pseudo-spin and valley edge states within single waveguiding channels, enhancing bandwidth while enabling multipath routing for THz information processing [38]. Parallel investigations of Bi_2_Se_3_ nanostructures show that rectangular antennas effectively confine Dirac plasmon polaritons to one-dimensional propagation paths, informing designs for directional quantum devices [39]. Systematic investigations of THz radiation from topological insulator Bi_2_Te_3_ nanofilms driven by femtosecond laser pulses have successfully realized the generation of efficient chiral THz waves with controllable chirality, ellipticity, and principal axis [40]. In addition, Bi_2_Se_3_ nanospheres exhibit Purcell factors exceeding 10^10^ for quantum emitters in the THz frequencies, suggesting strong nanoscale light–matter interaction capabilities [41].

**Fig. 2.**
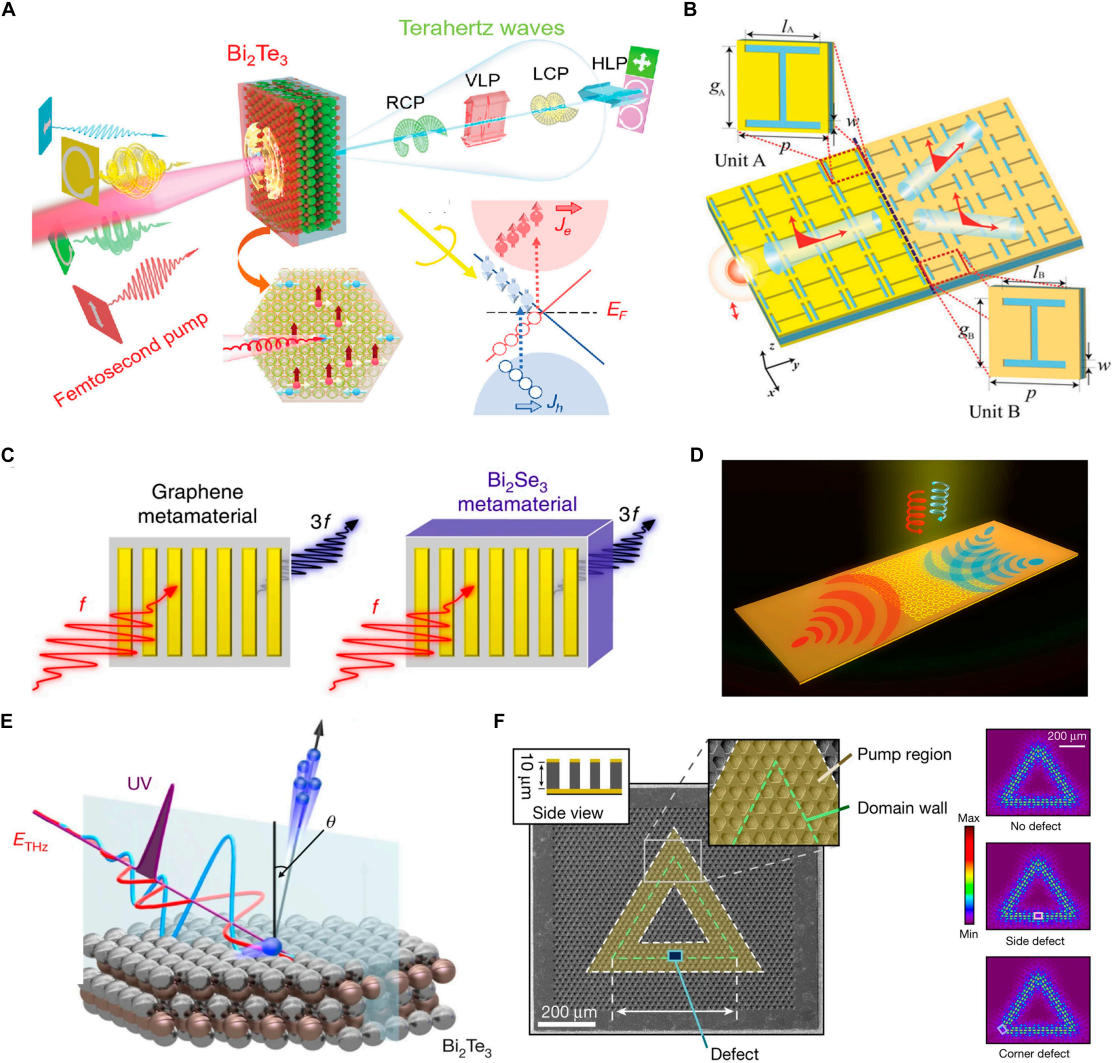
THz topological metadevices for free space applications. (A) 3D topological insulator Bi_2_Te_3_ [40]. (B) Anomalous wave propagation in topological transition metadevice [47]. (C) Milliwatt THz harmonic generation [48]. (D) Spin-decoupled wavefront manipulation [49]. (E) Subcycle observation of lightwave-driven Dirac currents [51]. (F) Design of a topological laser [52].

Recent advances in THz beam generation and manipulation have established diverse technical approaches [42]. Programmable Bessel beam generation at 0.3 THz has been achieved through 3D-printed axicon arrays, demonstrating applications in imaging and orbital angular momentum (OAM)-encoded communications [43]. Experimental verification confirms the self-healing characteristics of OAM-bearing THz Bessel beams (topological charges *l* = 3, 4) during propagation through dispersive media [44]. Complementary approaches employ phase-engineered diffractive elements combining spherical harmonic axicons with spiral plates to produce perfect vortex beams featuring tunable topological charges [45]. Parallel developments reveal ZnTe crystal-mediated THz vortex generation through infrared-to-THz topological charge transfer, establishing novel spectral conversion mechanisms [46]. Metasurface investigations demonstrate anomalous surface plasmon polariton propagation governed by eigenmode equal-frequency contour engineering [47]. Concurrently, topological insulator metamaterials exhibit enhanced THz harmonic generation efficiency, indicating transformative potential for advanced wireless communication systems [48].

The development of cascaded metadevices integrating helical plasmonic and dielectric metasurfaces has expanded design possibilities for photonic systems. The investigation of spin-decoupled wavefront manipulation of THz surface plasmons using freestanding topological metasurfaces has shown promise for expanding the control methods of surface plasmons and broadening the application scenarios of topological metadevices [49]. Further theoretical investigations examine nonparaxial mode diffraction in THz laser metal waveguide resonators, employing numerical modeling and Rayleigh-Sommerfeld vector theory to analyze mode interactions with spiral phase plates and vortex beam propagation in free space [50]. In a separate development, Reimann et al. [51] implement angle-resolved photoemission spectroscopy with subcycle time resolution, capturing THz light pulse-induced acceleration of Dirac fermions in Bi_2_Te_3_’s topological surface state. Complementing these findings, the fabrication sensitivity of quantum cascade lasers has been addressed through topological robustness, enhancing their utility as THz radiation sources [52].

THz topological metadevices exhibit superior performance in free-space systems, demonstrating capabilities for multimodal information routing, directional plasma propagation, and efficient chiral THz wave generation while enabling precise vortex beam manipulation and OAM transmission. Advancements in structural engineering and material optimization, including 3D-printed axicon arrays, ZnTe crystal integration, and topological insulator metamaterials, have facilitated breakthroughs in THz beam shaping, spectral conversion, and high-power harmonic generation. These innovations establish critical technological foundations for emerging applications in high-dimensional communications, precision THz imaging, and quantum state control.

## Reconfigurable THz Metadevices

Reconfigurable THz devices overcome the inherent limitations of conventional static architectures by integrating external stimuli-responsive mechanisms. Through precise modulation via electric fields, thermal gradients, or optical excitation, these adaptive systems enable real-time control of beam steering, phase manipulation, polarization conversion, and spectral characteristic, demonstrating unprecedented operational flexibility and multifunctional capabilities [7,53–56]. Specifically, compared with optical and thermal modulation methods, electrically modulated reconfigurable platforms have attracted wide interest due to their low cost, low energy consumption, and programmability. These dynamic platforms not only form the foundation for intelligent THz systems but also accelerate practical implementations in next-generation communications, real-time hyperspectral imaging, and advanced sensing technologies. Current reconfigurable THz devices fall into 4 categories: LC-based configurations, plasma-enhanced metadevices, and PCM-based architectures, and mechanically reconfigurable metadevices.

### LC-based platforms

The field-sensitive orientation of LC molecules enables dynamic control of their optical properties via external electric and magnetic field tuning, achieving THz wave modulation with millisecond-scale response times. LC-based reconfigurable THz devices have emerged as a focal point of research owing to their potential in next-generation wireless communications, high-resolution imaging, and sensitive detection applications [57–59]. These systems utilize the tunable birefringence and rapid response of LCs to dynamically control THz waves, with recent progress demonstrating 3 principal technical directions. First, beam steering architectures show important advancement: a reconfigurable intelligent surface achieves 20° to 60° beam scanning at 415 GHz through pixelated grating electrodes [60], while a programmable metasurface enables 2-dimensional beam shaping at 94 and 220 GHz through switchable phase states [61]. Further extending these capabilities, a phase gradient metasurface demonstrates 47.5° and 32.5° beam deflection at 0.69/0.94 THz with >97% modulation depth [62]. Beyond beam steering, polarization conversion provides an essential capability for advanced wavefront manipulation. A double-grating converter achieves <3 dB insertion loss at 388 to 426 GHz using Fabry-Perot resonance [63], complemented by a geometric-phase modulator maintaining >70% efficiency across 0.8 to 1.2 THz through orientation-engineered LCs [64]. These developments are augmented by a double split-ring metasurface attaining >90% conversion ratio over 2.55 to 7.61 THz through optimized notch structures [65].

**Fig. 3.**
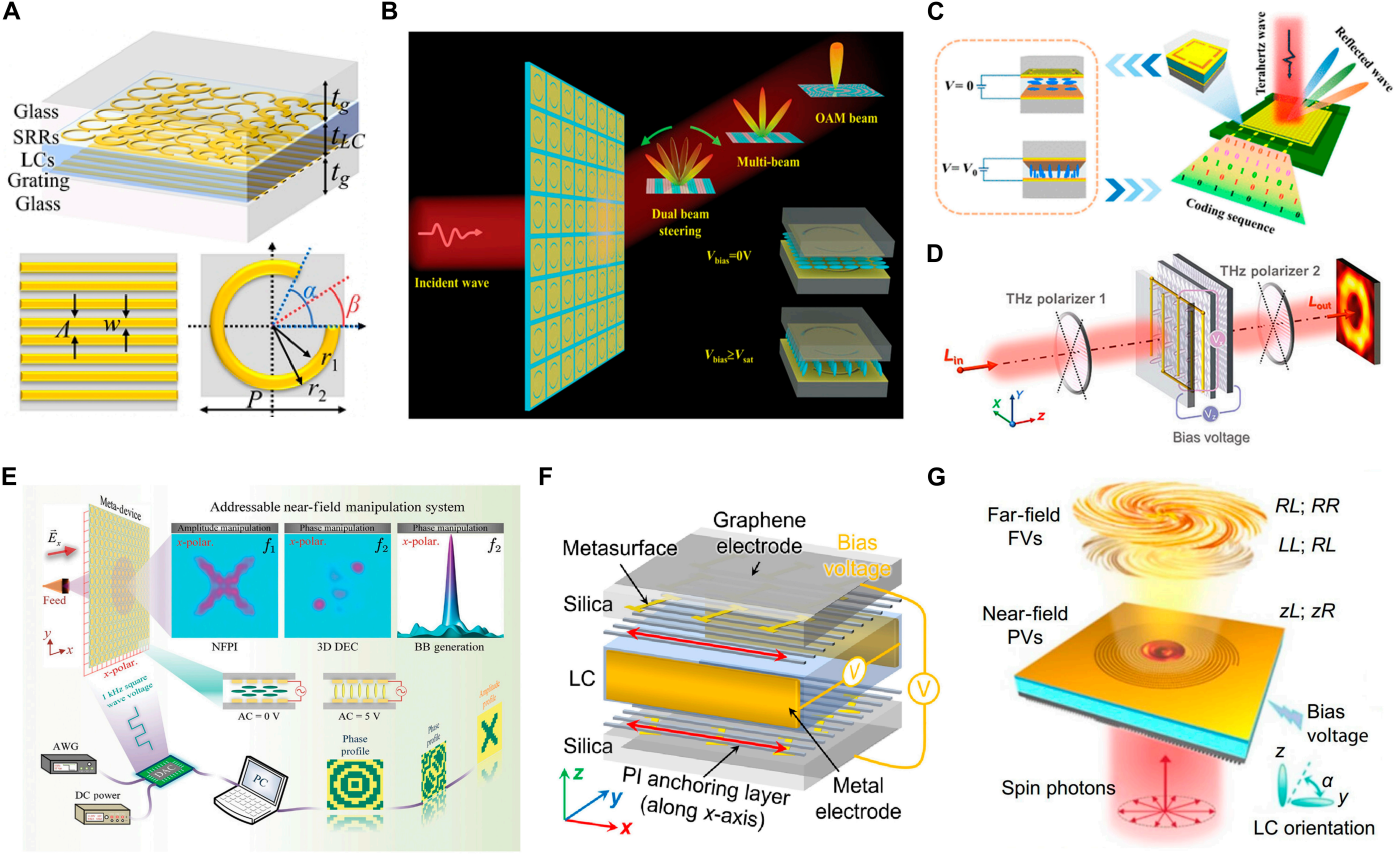
LC-based reconfigurable THz metadevices. (A) Phase gradient metasurface with liquid crystal-enhanced cavity mode conversion [62]. (B) Transmissive digital coding metasurfaces based on liquid crystals [66]. (C) LC integrated programmable metasurfaces [67]. (D) Vortex vector beam conversion [69]. (E) Individually addressable transmissive metadevice [68]. (F) Flexible manipulation enabled by anisotropic liquid crystal coupled chiral metasurfaces [71]. (G) Dielectric-liquid crystal-plasmonic metadevice [75].

This field continues to progress through innovations in programmable metasurfaces. A 1-bit transmissive metasurface enables dual-beam steering via Fano resonance modulation [66], while its reflective counterpart achieves a 180° phase shift at 0.675 THz, allowing wide-angle beam control [67]. Building on these advances, a 16 × 16 addressable metadevice delivers over 90% amplitude modulation for near-field imaging applications [68]. Material integration strategies further enhance functionality. A cascaded metadevice, for example, enables vortex-vector beam conversion through asymmetric phase modulation [69], and a 108-GHz reflectarray achieves ±40° beam steering through scalable LC integration [70]. In parallel, chiral metadevices that combine anisotropic metasurfaces with LCs exhibit circular dichroism of 33 dB at 0.94 THz [71]. Performance optimization remains a key focus. Metal-insulator-metal metasurfaces serve as tunable filters with a 10% spectral shift [72], aided by interdigitated electrodes that shorten LC response times by 65% [73]. Furthermore, inserting a nematic LC layer between the grating and resonant structure enables 32 distinct reflected wave coding states, spanning 0° to 235°, through voltage-controlled molecular reorientation [74]. LC-based platforms have also demonstrated the generation of plasmonic and free-space vortices, achieving average mode purities above 85%, underscoring their potential in THz communication and information processing [75].

LC-based THz reconfigurable metasurfaces demonstrate considerable promise for imaging and communication systems, yet their practical deployment encounters several technical hurdles. Performance constraints stem from ohmic losses in metallic structures and surface irregularities in dielectric components, necessitating the investigation of advanced materials such as ceramics and ferromagnetic compounds. The development of innovative LC formulations, particularly ferroelectric nematics, coupled with detailed studies of LC-metasurface interactions, is crucial for improving device capabilities [76]. Furthermore, the fabrication process remains challenging due to its complexity and expense, highlighting the need for cost-effective large-scale production methods and enhanced integration technologies. Future investigations should concentrate on optimizing device performance through material innovation, structural design refinement, and manufacturing process improvements, thereby enabling broader application of these dynamically tunable devices.

### Plasma-based metadevices

Recent advances in THz plasma metadevices demonstrate promising developments. A silicon plasma antenna employing vertical positive-intrinsic-negative (PIN) diode arrays demonstrates >5 dBi realized gain at 27.5 to 29.6 GHz through vertical plasma beam steering, addressing conductivity constraints of surface-type architectures [77]. Reconfigurable microcavity plasma photonic crystals achieve tunable electromagnetic responses across 100 GHz to 1 THz, with a 157-GHz prototype showing narrow stopband characteristics [78]. Voltage-controlled plasma metamaterial absorbers enable polarization-insensitive broadband tuning via selective resonator activation [79]. Dual-modulated vanadium dioxide/graphene heterostructures exhibit a 0.95 transmission coefficient with a 27.6-ps group delay, demonstrating potential for THz slow-light manipulation [80]. All-dielectric microwave plasma generators further suggest low-loss solutions for high-frequency applications [81]. In addition, a THz modulator based on a laser-driven plasma can amplify or attenuate an incident THz wave of 0.1 to 2.0 THz within a few picoseconds by adjusting its dipole phase [82].

The modeling and analysis of microplasmas have yielded important insights into their behavior across various frequencies. Resonant regions emerge when the excitation frequency matches the local plasma frequency [83]. A Weibull analysis of atmospheric pressure plasma generation reveals that fabrication methods and materials independently influence both the *Q*-factor of the resonators and the breakdown voltage. The power requirements for breakdown vary considerably based on these factors [84]. The gate-voltage tunability of plasmons in single-layer graphene structures has been investigated, quantifying the effects of gate voltage and gate dielectric thickness on plasmon propagation characteristics in graphene [85]. Furthermore, a reconfigurable electromagnetically induced transparency metamaterial that simultaneously couples with incident electric and magnetic fields has been proposed. This structure demonstrates low loss, polarization insensitivity, and slow-wave effects, indicating potential applications in communication and sensing technologies [86]. In particular, atmospheric plasma-based approaches utilize femtosecond laser-induced atmospheric plasma filaments, which enable THz pulse integration via epsilon-near-zero (ENZ) region confinement [87].

The plasma platform generates regions of high carrier density in semiconductors/gases through electrical injection, electric field breakdown, or ultrafast laser pumping, enabling resonance coupling between the plasma frequency and the structure’s resonance frequency. This achieves sub-nanosecond-level gain, absorption, or phase modulation of THz waves. However, this method faces numerous challenges [88]. First, triggering plasma requires high voltages or high-power pulses, leading to thermal management and energy consumption issues. Second, the uniformity of microplasma size/density affects device consistency. Additionally, material fatigue and lifetime degradation caused by repeated breakdown have not been fully resolved, and overall system integration remains relatively complex. Moreover, plasma array homogeneity is also one of the key factors affecting device performance, which can be further improved by precise temperature control.

### PCMs-based metadevices

PCMs have emerged as critical components for reconfigurable THz devices, leveraging their structural phase transitions between amorphous and crystalline states to enable dynamic THz wave modulation for advanced imaging, communication, and sensing systems. Vanadium dioxide (VO₂) demonstrates particular promise through its thermally induced insulator-to-metal transition near 68 °C, with engineered VO₂-based devices achieving 20 dB transmission contrast and >90% rejection efficiency in reconfigurable THz filters [89]. Recent innovations include hybrid VO₂ metasurfaces that achieve spectral window shifting [90] and thermally tunable ultra-broadband THz absorbers [91]. Concurrently, silicon- and germanium-based architectures show complementary potential for THz logic operations [92] and active wavefront manipulation [93], collectively expanding the toolkit for adaptive THz photonics.

Ge₂Sb₂Te₅ (GST), a leading PCM, has attracted considerable interest due to its non-volatile and reversible phase transition characteristics [94]. Recent demonstrations of optically tunable, ultrafast broadband THz polarimetric devices utilizing GST highlight the adaptability for dynamic THz applications [95]. Important progress has been achieved in developing non-volatile reconfigurable THz metadevices [96–98] and wide-angle broadband THz antireflection coatings [99] based on this material system. These developments collectively demonstrate the capacity of PCMs to enable multifunctional THz technologies. Notably, GST-engineered THz metamaterials show particular promise for 6G communication systems, as evidenced by comprehensive reviews addressing both current technical limitations and future research priorities. Complementary advances include dynamically reconfigurable platforms integrating chalcogenide metasurfaces [100] and electrically addressable intelligent THz metadevices [101], further expanding PCM-driven innovation in this field. While PCM-based THz devices demonstrate substantial application potential, key challenges persist in optimizing switching speeds and minimizing power requirements [102–107]. Continued research on PCMs is expected to yield transformative THz technologies with broad implications across scientific and industrial applications.

Phase-change platforms leverage materials such as VO₂ and GST, which exhibit reversible transitions between crystalline and amorphous states (or insulating and metallic states), accompanied by differences in conductivity and refractive index. This enables non-volatile amplitude and phase modulation, broadband absorption, or frequency window shifting in the THz region. Similarly, the main challenges facing the large-scale application of this method in the THz band include the following: phase change switching, especially the crystallization process, still consumes a large amount of energy and heat; PCMs exhibit cyclic fatigue from repeated phase transitions and, under specific environmental conditions, may undergo chemical interactions leading to progressive property degradation or functional failure; and nanoscale patterning and large-area uniform deposition processes are technically challenging. In addition, how to further improve sub-nanosecond speed and reduce drive voltage is key to the advancement of PCM devices toward 6G communications and real-time imaging applications.

### Mechanically reconfigurable metadevices

In contrast to above solutions that require active stimuli, Moiré meta-devices provide a novel approach to dynamically modulate electromagnetic waves. Interlayer rotation, twist, or lateral displacement creates synthetic phase gradients through purely geometric manipulation, bypassing external stimuli. This mechanical degree of freedom enables power-free, continuous modulation of optical properties (amplitude, phase, and polarization) across broadband spectra. For example, THz Moiré lenses achieve simultaneous imprinting of distinct topological charges on orthogonal circular/linear polarizations while dynamically adjusting focal length from 9 to 3 mm with <6% error [108]. Similarly, twisted dielectric axicons enable dynamic control of Bessel beam order and non-diffracting range [109], while bilayer twisted elements demonstrate reconfigurable functionality as varifocal lenses and polarization filters, maintaining >35% efficiency through 150° rotational displacement [110]. Further innovations include vector–vortex beam generators with wavelength-scale focal steering (<10% radial symmetry deviation) [111] and Bessel beams featuring concurrent topological charge tuning and spatially variant polarization engineering [112]. However, these platforms suffer from limited response speed and scalability due to the inherent problems of reconfigurable approaches based on mechanical control.

Reconfigurable THz metadevices based on LCs, plasmas, and PCMs offer complementary approaches to dynamic wavefront control, each with distinct trade-offs. LC-based systems exploit voltage-driven molecular reorientation to enable tunable anisotropy, supporting beam steering, polarization conversion, and OAM generation via programmable metasurfaces. Despite advantages such as low-voltage operation and high modulation depth, their practical deployment is limited by slow response times and fabrication complexity. Plasma-based devices achieve sub-nanosecond modulation through ultrafast carrier dynamics, triggered by electrical breakdown or optical excitation, yielding slow-light effects and ENZ field confinement. However, challenges including high power dissipation, microplasma inhomogeneity, and reliability concerns hinder their integration. PCM platforms, which rely on reversible phase transitions in VO₂ or GST, provide nonvolatile, broadband control over THz amplitude and phase, enabling dynamic filtering and logic operations. Their scalability and switching speeds are constrained by thermal fatigue, crystallization energy barriers, and demanding nanofabrication requirements. Together, these tunable THz technologies hold potential for 6G communications, real-time imaging, and adaptive sensing, though further progress in materials engineering, switching architectures, and scalable fabrication remains critical.

**Fig. 4.**
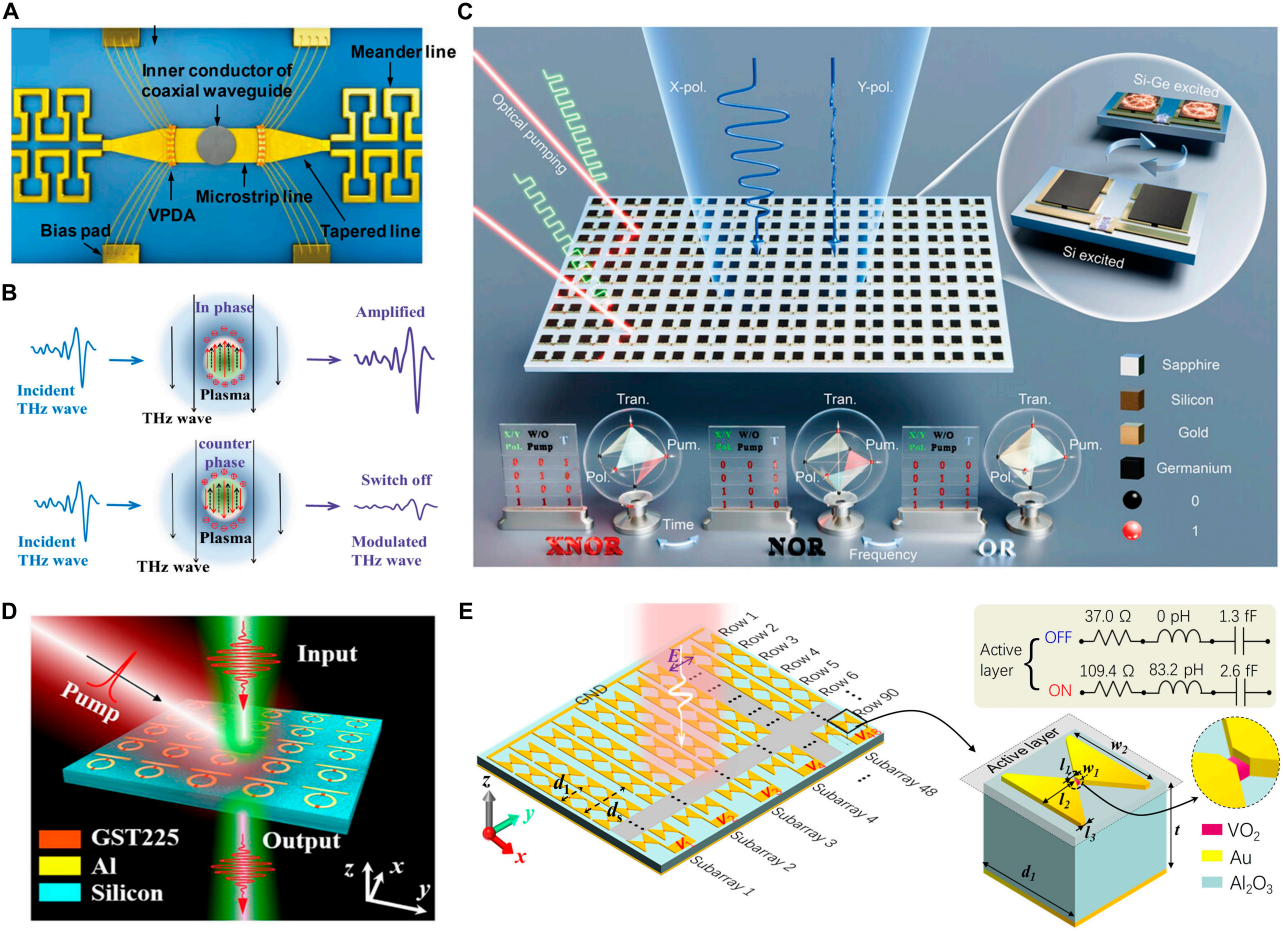
Plasma and PCMs-based THz reconfigurable metadevices. (A) Reconfigurable beamforming silicon plasma antenna [77]. (B) Gate-voltage tunability of plasmons [82]. (C) Reconfigurable electromagnetically induced transparency metamaterial [92]. (D) Reversible switching of the metasurface-induced transparency in the THz spectrum [100]. (E) Electrically addressable integrated intelligent THz metasurface [101].

## THz Reconfigurable Topological Metadevices

Over the last few years, the development of THz reconfigurable topological components facilitates the enhancement of the functionality of next-generation communication, sensing, and imaging systems. For free space application, synthetic antiferromagnet systems demonstrate reconfigurable chiral THz emission for nanoscale spintronic analysis [113], while complementary developments in Dirac semimetals produce polarization-independent coding metasurfaces enabling broadband beam control and OAM manipulation [114]. Additionally, studies for Bi₂Se₃ also reveal tunable Dirac plasmon interactions that enable nonlinear optical components [115]. PCMs have enabled nonvolatile silicon chips to demonstrate stable resonance modulation through multi-level reconfiguration, as shown in devices employing GST [116]. Electrically tunable solutions achieve dynamic control, exemplified by photonic notch filters with >20 dB suppression depth and precise frequency tuning [117]. LC integration extends reconfiguration capabilities through electric-field manipulation of topological edge states in valley photonic crystals [118], with reflective metasurfaces implementing 2-bit coding for real-time beam steering and vortex generation at 0.67 THz [119].

**Fig. 5.**
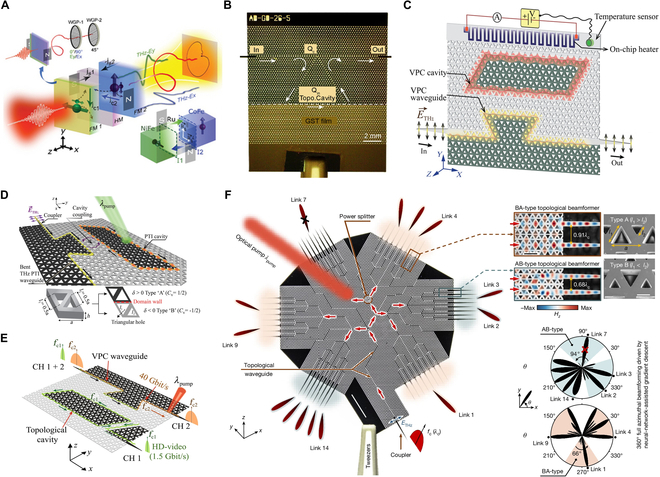
THz reconfigurable topological metadevices. (A) Reconfigurable chiral spintronic THz emitters [113]. (B) Non-volatile reconfigurable phase change topological photonics [116]. (C) Electrically tunable topological notch filter [117]. (D) Ultrahigh-Q THz topological cavities on a chip [120]. (E) Phototunable chip-scale topological photonics [121]. (F) On-chip topological beamformer [122].

The advancements in reconfigurable chip-scale THz topological devices also mark important progress toward 6G communication. A notable study achieved an ultrahigh-*Q* (0.2×10^6^) THz topological cavity on a chip, leveraging a CMOS-compatible platform for efficient optical control and modulation [120]. Another breakthrough introduced a phototunable THz topological device, incorporating a 160-Gbps waveguide and demultiplexer [121], which demonstrated exceptional channel isolation and real-time data transmission capabilities. Moreover, on-chip reconfigurable topological devices leveraging valley vortices achieve 72 Gbps wireless transmission across 300 mm and support multi-channel 40 Gbps operation [122], demonstrating compatibility with terabit-scale 6G/XG systems based on massive multiple-input multiple-output (MIMO) architectures. These studies underscore the potential of silicon-based topological photonic crystals in enhancing device performance, minimizing crosstalk, and enabling active tunability, thereby advancing the development of THz integrated circuits and 6G technologies. Notably, compared to microwave devices, THz metadevices feature larger bandwidths, better directionality, and a more compact volume.

## Conclusion

THz topological metasurfaces exhibit efficiency and ease of integration for the manipulation of THz waves. However, traditional topological metadevices are inherently static, lacking the capacity for structural or electromagnetic reconfiguration, thus limiting their scalability. The introduction of tunable mechanisms into the design of THz topological devices addresses this limitation, enabling dynamic programmability, reduced loss, and enhanced robustness. By integrating electro-tunable materials, PCMs, and active response mechanisms such as ferroelectric and photo-induced charge carriers, researchers have preliminarily achieved external control over topological band structures, edge state transport properties, and even the topological phase itself. These advances redefine the design space of THz photonics and pave the way for next-generation reconfigurable THz communications, tunable filters, and non-reciprocal photonic devices [123–126].

Nevertheless, reconfigurable topological THz metadevices still face multiple technical barriers that need to be addressed. Future research will focus on 2 complementary strategies: on the one hand, the development of novel materials with enhanced switching dynamics and spectral response properties to improve THz frequency reconfigurability while reducing energy dissipation, and on the other hand, autonomous optimization of device architectures and adaptive control of signal modulation parameters through the integration of machine learning algorithms. Furthermore, the development of a novel advanced THz reconfigurable topological platform requires synergistic advances in materials innovation and fabrication processes, especially nanofabrication technology, which is crucial to achieve the complex geometries required for efficient electromagnetic wave manipulation.
